# Case Report: Anti-N-Methyl-D-Aspartate Receptor Encephalitis Manifesting With an Isolated Psychiatric Episode and Normal Ancillary Tests

**DOI:** 10.3389/fpsyt.2022.905088

**Published:** 2022-06-02

**Authors:** Denis Pavǎl, Claudia Mihaela Cîmpan, Nicoleta Gherghel, Laura Otilia Damian, Nicoleta Tohǎnean, Ioana Valentina Micluţia

**Affiliations:** ^1^Department of Psychiatry, “Iuliu Haţieganu” University of Medicine and Pharmacy, Cluj-Napoca, Romania; ^2^Psychiatry Clinic, Emergency County Hospital, Cluj-Napoca, Romania; ^3^Neurology Clinic, Emergency County Hospital, Cluj-Napoca, Romania; ^4^Department of Rheumatology, Center for Rare Musculoskeletal Autoimmune and Autoinflammatory Diseases, Emergency County Hospital, Cluj-Napoca, Romania; ^5^Department of Neurology, “Iuliu Haţieganu” University of Medicine and Pharmacy, Cluj-Napoca, Romania

**Keywords:** autoimmune encephalitis, autoimmune psychosis, NMDA, neuronal antibodies, isolated, case report

## Abstract

The majority of patients with anti-N-Methyl-D-Aspartate receptor (NMDAR) encephalitis present with psychiatric symptoms and subsequently develop neurological features. However, isolated psychiatric episodes occur in <5% of affected individuals, less frequent at disease onset (<1%) compared to relapse (4%). We report the case of a previously healthy 24-year-old female who presented with psychotic symptoms and behavioral alterations. Despite therapy, she showed no improvement and subsequently developed catatonic features. While the ancillary tests were normal, the clinical warning signs raised the suspicion of anti-NMDAR encephalitis which we later confirmed. Given its strong association with underlying tumors, we screened the patient and found an ovarian teratoma. Once removed, the patient displayed a substantial improvement in the mental status. Besides being extremely rare, this case illustrates the need to maintain clinical suspicion of anti-NMDAR encephalitis even in the absence of neurological features or paraclinical anomalies.

## Introduction

Anti-N-Methyl-D-Aspartate receptor (NMDAR) encephalitis is a neuroinflammatory disorder in which antibodies target the GluN1 subunit of the receptor and interfere with its function (1). While >85% of affected individuals present with psychiatric symptoms (2), most eventually develop neurological symptoms (autonomic dysfunction, seizures, and movement disorders) which facilitate the diagnosis (1). Nevertheless, uncommon isolated psychiatric episodes have been described in 5% of affected individuals, though less frequent at disease onset (<1%) compared to relapse (4%) (3, 4).

While a definite diagnosis of anti-NMDAR encephalitis requires the presence of IgG antibodies against the GluN1 subunit of the NMDAR in CSF (5), other CSF anomalies along with MRI and EEG abnormalities help orienting the diagnosis (1). Moreover, 38% of patients overall and 46% of female patients have underlying tumors, mostly (94%) ovarian teratomas (6) which have been shown to contain neural tissue expressing NMDAR (1). Appropriate and timely treatment, such as immunotherapy and tumor removal, leads to substantial improvement and positive outcomes in most patients with anti-NMDAR encephalitis (6).

In this paper, we report the case of a young female who presented with psychotic symptoms and behavioral alterations. While she displayed no neurological alterations and had normal ancillary tests, we established a diagnosis of anti-NMDAR encephalitis owing to a high index of suspicion. Subsequently, we screened the patient and found an ovarian teratoma. Tumor removal led ultimately to a rapid and substantial recovery of the patient. While extremely rare, this case illustrates the importance of maintaining clinical suspicion of anti-NMDAR encephalitis even in the absence of neurological features or paraclinical anomalies.

## Case Presentation

A 24-year-old female patient presented to the Psychiatry Emergency Department with delusions of pregnancy, visceral hallucinations and bizarre behavior, as she stated repeatedly that she was pregnant and about to give birth. The patient's complaints started a few hours prior to the presentation. As soon as symptoms developed, she requested an emergency gynecological evaluation which ruled out pregnancy. Apart from seeming “absent-minded” at work earlier during that day, the patient had no prodromal symptoms. She had unremarkable personal and family history of mental disorders. Besides having non-autoimmune hypothyroidism for which she received hormone replacement therapy, her past medical history was not significant. No history of drug abuse was noted.

On presentation, the patient was restless, fearful and exhibited bizarre behavior. She was covering her eyes and ears and refused to answer to most questions. Physical exam was within normal limits. The basic laboratory analyses and brain CT scan were normal. Based on these findings, the patient was admitted to the psychiatric ward and started on antipsychotics and adjunctive benzodiazepines. Despite being on a combination of haloperidol and olanzapine, the patient did not show any sign of improvement over the next 2 weeks. She displayed frequent episodes of disorganized behavior with agitation and aggression and continued to state that she was pregnant and about to give birth. She also reported short-term memory deficits which were difficult to confirm due to the severity of psychotic symptoms.

On day 15 of the admission (D15), the patient was referred to a neurologist. While the neurological exam was normal, the persistence of psychotic symptoms despite therapy raised the suspicion of an organic psychosis. Thus, the patient underwent a screening work-up including brain-associated systemic antibodies, vitamin levels, rheumatic markers, and pathogens (see Table 1). In addition, the patient had an EEG which showed numerous artifacts (as she was uncooperative and restless), yet no slowing or epileptiform discharges. Moreover, she underwent a brain MRI under anesthesia which was also normal.

**Table 1:** Paraclinical findings in anti-NMDAR encephalitis manifesting with an isolated psychiatric episode [adapted from the screening approach proposed by Endres et al. (7)].

Vitamins	Folic acid (B_9_)	Low (4.88 ng/ml, ref. range: 5,9–23,2 ng/ml)		
	Cobalamin (B_12_)	Normal		
Pathogens	Serologies for Lyme disease, syphilis, HIV, hepatitis, toxoplasmosis, EBV, CMV, HSV, VZV	Normal		
Immunological serum screening	Rheumatic/immunological markers (IgA/IgM/IgG, C3, C4)	High C3 (1.88 g/l, ref. range 0.9–1.8 g/l) High C4 (0.622 g/l, ref. range 0.1–0.4 g/l)		
	Potential antineuronal-rheumatic antibodies (ANA, p- & c-ANCAs, anti-phospholipid, anti-Ro, anti-cardiolipin)	Normal		
	Brain-associated systemic antibodies (anti-TG antibodies, anti-TPO antibodies)	Normal		
	Neuronal IgG antibodies against cell surface antigens (NMDAR)	High (1:2560, ref. range: <1:10)		
	Neuronal IgG antibodies against intracellular antigens (Yo, Hu, CV2/CRMP5, PNMA2/Ta, Ri, recoverin, SOX1, amphiphysin, titin)	Normal		
Cerebrospinal fluid analysis	Basic analyses (WBC count, total protein, albumin quotient, IgG index, OCBs)	Normal		
	Neuronal IgG antibodies against cell surface antigens (NMDAR)	High (1:32, ref. range: <1:1)		
	Infectious and other markers (bacterial, fungal & BioFire multiplex PCR for microbial detection, cytopathology)	Normal		
Instrument-based diagnostics	Resting state EEG	Normal (despite artifacts)		
	Brain CT & MRI (with contrast agent)	Normal		
	CT thorax, abdomen and pelvis & pelvic MRI (with contrast agent)	Right ovarian mass suggestive for a mature cystic ovarian teratoma		
Neuropsychological testing		**Pre-surgery**	**Post-surgery**	**1-month follow-up**
	PANSS (total score)	136	47	32
	MoCA	18	23	27

*EBV, Epstein-Barr virus; CMV, cytomegalovirus; HSV, herpes simplex virus; VZV, varicella-zoster virus; C3, complement component 3; C4, complement component 4; ANA, antinuclear antibodies; ANCA, antineutrophil cytoplasmic antibodies; TG, thyroglobulin; TPO, thyroid peroxidase; NMDAR, N-methyl-D-aspartate receptor; CRMP5, collapsin response mediator protein 5; PNMA2, paraneoplastic antigen Ma2; OCB, oligoclonal bands; PANSS, Positive and Negative Syndrome Scale; MoCA, Montreal Cognitive Assessment*.

On D29, the patient began to exhibit catatonic symptoms, such as mutism, mannerisms and echolalia. While the symptoms subsided rapidly after receiving 6 mg of intravenous lorazepam daily, she continued to display psychotic symptoms, as well as behavioral alterations. Given the persistent psychotic symptoms despite therapy along with catatonia, we raised the suspicion of anti-NMDAR encephalitis. Thus, a serum NMDAR IgG antibody analysis was performed with a fixed cell-based assay using EU90 cells transfected with the GluN1 subunit of the NMDAR complex (Euroimmun, Germany). On D42, the results came back positive (antibody titer of 1:2560, reference range <1:10). After a multidisciplinary discussion with neurologists and rheumatologists, the patient underwent a lumbar puncture for CSF analysis, NMDAR antibodies included. While awaiting the results, on D48 she was started on prednisone 60 mg daily.

**Figure 1:**
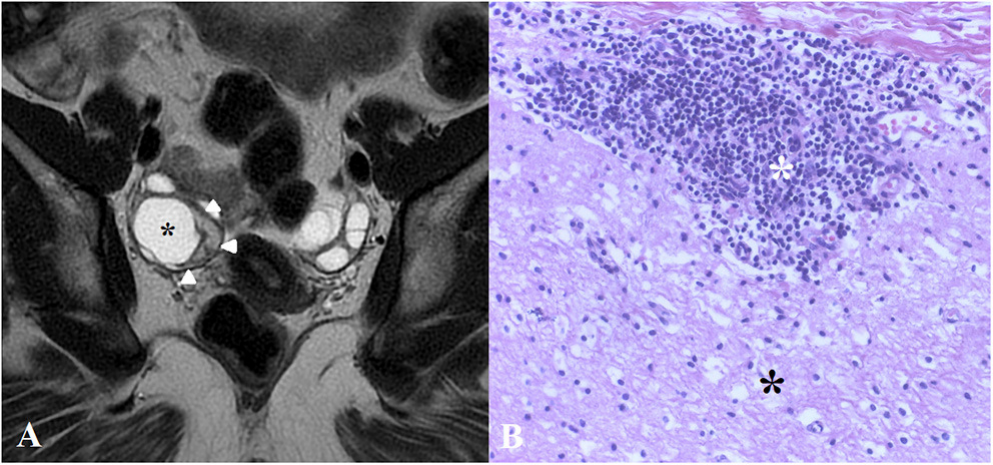
**(A)** Coronal T2-weighted image shows a mature cystic teratoma in the right ovary. At the periphery of the cystic space (black asterisk), there is a crescent-shaped solid component (white arrowheads). **(B)** Close contact between neural tissue (black asterisk) and dense lymphoid aggregate (white asterisk) on hematoxylin & eosin stain (×20). This aspect was observed on a section of the crescent-shaped solid component presented in **(A)**.

Given the paraneoplastic association, we performed a CT scan of the thorax, abdomen and pelvis which revealed a right ovarian mass suggestive for an ovarian teratoma; the pelvic MRI (with contrast) confirmed the aspect of a right mature cystic ovarian teratoma (shown in Figure 1A) for which she was scheduled for surgery. In the meantime, on D57 we confirmed the diagnosis of anti-NMDAR encephalitis as the patient had a high CSF antibody titer (1:32, reference range <1:1), while no other CSF anomalies were detected (see Table 1). Around this time, the patient started to show improvement of the psychotic symptoms which in turn emphasized her cognitive deficits.

On D60, the patient was transferred to the gynecologic ward where she underwent a right adnexectomy. The histopathological exam confirmed the mature ovarian teratoma, highlighting dense lymphocyte infiltration near neural tissue shown in Figure 1B. 4 days later, she returned on the psychiatric ward displaying a substantial improvement in the mental status. While the psychotic symptoms ceased completely, short-term memory and attention deficits became increasingly obvious (see Table 1, Neuropsychological testing). We began to progressively taper the prednisone and initiated oral azathioprine 150 mg daily in order to maintain remission; at the same time, we began to taper the antipsychotics. All this time, the patient remained admitted in the psychiatric ward, as she did not develop any signs of neurological dysfunction. On D67, the patient was discharged and further planned for a multidisciplinary follow-up.

At 1-month follow-up, the patient displayed only mild short-term memory deficits which had no impact on her daily activities. She had normal neurological status, no psychotic symptoms and displayed near-normal cognition, while the serum NMDAR antibodies were negative. She tolerated azathioprine and was being slowly tapered off prednisone under close rheumatological monitoring. The patient had little recollection of the admission period and planned to return to work as soon as possible. At 2-months follow-up, the patient was virtually symptom-free and returned to work without experiencing major difficulties. She was being slowly tapered off both prednisone and azathioprine. At 3-months follow-up, the patient was symptom- and medication-free, while continuing to work without further issues. In Table 2, we summarized the timeline of relevant events during the patient's episode of care.

**Table 2:** The timeline of relevant events during the episode of care.

**Timeline**	**Relevant event**
D1	The patient was admitted due to psychosis with behavioral alterations. Physical exam, basic laboratory analyses and brain CT scan were normal.
D2-D14	Despite antipsychotic therapy, the patient continued to exhibit psychotic symptoms with behavioral alterations, along with short-term memory deficits.
D15	The patient was referred to a neurologist due to suspicion of an organic psychosis. However, the neurological exam, screening work-up, EEG, and MRI were unremarkable.
D29	The patient started exhibiting catatonic symptoms which raised suspicion regarding anti-NMDAR encephalitis.
D42	We detected a high serum level of anti-NMDAR IgG antibodies and performed a lumbar puncture.
D48	The patient was started on prednisone 60 mg daily. Besides, she was found to have an ovarian teratoma and was scheduled to surgery.
D57	We confirmed the anti-NMDAR encephalitis as the patient had a high CSF antibody level. She began to show improvement of the psychotic symptoms which emphasized her cognitive deficits.
D60	The patient underwent an adnexectomy in order to remove the ovarian teratoma.
D64	The patient was no longer psychotic, but displayed short-term memory and attention deficits. While tapering the prednisone, she was initiated oral azathioprine 150 mg daily.
D67	The patient was discharged.
1-month follow-up	The patient had only mild short-term memory deficits, while having little recollection of the admission period.
2-months follow-up	The patient was symptom-free and returned to work without experiencing major difficulties, while tapering both prednisone and azathioprine.
3-months follow-up	While continuing to work, the patient was symptom- and medication-free.

*NMDAR, N-Methyl-D-Aspartate receptor; CSF, cerebrospinal fluid*.

## Discussion

The diagnosis of anti-NMDAR encephalitis is not always straightforward, as uncommon presentations occur. Our patient displayed not only isolated psychiatric features, but also normal findings on ancillary tests. In a cohort of 571 patients with anti-NMDAR encephalitis, only 5 (0.9%) individuals had isolated psychiatric episodes at disease onset (3). However, all of these 5 patients had MRI anomalies which prompted NMDAR antibody testing; besides, 2/5 had abnormal EEG, while 4/5 had CSF anomalies. In our case, only a high index of suspicion led to a more comprehensive investigation including NMDAR antibody testing.

Studies so far have failed to emphasize a specific psychiatric phenotype in anti-NMDAR encephalitis (2). However, one study argues that its psychiatric features meet the criteria for cycloid psychosis (CP). While not specific for anti-NMDAR encephalitis, CP delineates acute psychotic episodes with a fluctuant clinical pattern mostly characterized by confusion, psychosis, movement disorders (including catatonia) and oscillations of mood (8). Our patient displayed a triad of behavior alterations (with agitation and aggression), psychosis and catatonia. Along with mood and sleep anomalies, these 5 symptom domains are among the most common reported clusters of psychopathological symptoms in anti-NMDAR encephalitis (4). As illustrated by our case, these psychiatric features develop rather sudden (1), while primary psychoses tend to have a more insidious onset. The course of the disease also differs; while psychiatric symptoms in anti-NMDAR encephalitis tend to fluctuate (8), they remain more stable in primary psychoses. Additionally, as in our case, the psychotic symptoms tend to be treatment-resistant which often require higher doses or combinations of antipsychotics (9). Thus, the sudden onset and peculiar course of the disease, the persistence of psychotic symptoms despite therapy along with the development of catatonia led us to consider the possibility of anti-NMDAR encephalitis.

Our patient did not show signs of neurological involvement, even though both ovarian teratomas (10) and high NMDAR antibody titres (11) are associated with severe neurological features. One might argue that the patient would have developed neurological symptoms later in the course of the illness. However, she started receiving corticosteroids 7 weeks after symptom onset which is beyond the expected window of neurological deterioration which often occurs within 2–3 weeks of psychiatric symptoms (3). Furthermore, despite being treated with a potent combination of haloperidol and olanzapine, the patient did not display any sign of antipsychotic intolerance which is frequently reported in patients with anti-NMDAR encephalitis (12).

While corticosteroids led to an initial improvement, tumor removal has proven key in the patient's recovery as lymphocyte infiltration near neural tissue pointed to the site of antigen presentation (13). Thus, the patient no longer had detectable serum NMDAR antibodies at 1-month follow-up. While having only mild memory deficits, she was not fully recovered. Yet, in accordance with the better prognosis and lower rate of relapse in individuals with underlying ovarian teratomas (6), the patient displayed a complete and sustained recovery at the 3-months follow-up.

We consider this case to be unusual for two reasons. First, the patient had isolated psychiatric features at disease onset which occurs in <1% of individuals with anti-NMDAR encephalitis (3). Second, the patient had normal findings on ancillary tests; MRI, EEG (despite artifacts, no clear anomaly was noted), and CSF analysis were unremarkable. So far, there is only one case report which reported similar findings (14). Some authors consider that such cases are sufficiently different from typical autoimmune encephalitis (AE) in order to establish a new concept of so-called “autoimmune psychosis” (AP) with its own diagnostic criteria (15). However, studies show that the only patients with first-episode psychosis having CSF anti-NMDAR antibodies are those at an initial stage of anti-NMDAR encephalitis or those who have a monosymptomatic form of AE (2). Outside this scenario, studies so far have failed to highlight other psychiatric populations which might harbor CSF anti-NMDAR antibodies. Regardless of the ongoing debate (2), our patient did not fulfill criteria for either “probable” AE (5) or “probable” AP (15). While a recent study addressed the existing gaps by including patients with isolated psychiatric symptoms in the diagnostic process (16), a high index of suspicion while looking for warning signs (17) remains key in making the right diagnosis.

## Data Availability Statement

The raw data supporting the conclusions of this article will be made available by the authors, without undue reservation.

## Ethics Statement

Ethical review and approval was not required for the study on human participants in accordance with the local legislation and institutional requirements. The patients/participants provided their written informed consent to participate in this study. Written informed consent was obtained from the individual(s) for the publication of any potentially identifiable images or data included in this article.

## Author Contributions

DP drafted the manuscript, conceived the theoretical framework, and analyzed the clinical data. CMC managed the case and analyzed the clinical data. NG helped with the theoretical framework, analyzed the clinical data, and worked on the manuscript. LOD provided rheumatological consultancy and analyzed the clinical data. NT provided neurological consultancy and analyzed the clinical data. IVM supervised the project, provided feedback, and revised the manuscript. All authors approved the final version of the manuscript.

## Conflict of Interest

The authors declare that the research was conducted in the absence of any commercial or financial relationships that could be construed as a potential conflict of interest.

## Publisher's Note

All claims expressed in this article are solely those of the authors and do not necessarily represent those of their affiliated organizations, or those of the publisher, the editors and the reviewers. Any product that may be evaluated in this article, or claim that may be made by its manufacturer, is not guaranteed or endorsed by the publisher.
